# Adaptation and acclimation of traits associated with swimming capacity in Lake Whitefish (*coregonus clupeaformis*) ecotypes

**DOI:** 10.1186/s12862-016-0732-y

**Published:** 2016-08-11

**Authors:** Martin Laporte, Anne C. Dalziel, Nicolas Martin, Louis Bernatchez

**Affiliations:** 1 Institut de Biologie Intégrative et des Systèmes (IBIS), Université Laval, Québec, Canada; 2 Department of Biology, Saint Mary’s University, Halifax, NS Canada; 3 School of Medicine, University of Wollongong, Northfields Avenue, Wollongong, NSW 2522 Australia

**Keywords:** Phenotypic plasticity, Local adaptation, Ecophysiology, Geometric morphometrics, Speciation, Parallel evolution, Swimming cost, *Salmonidae*

## Abstract

**Background:**

Improved performance in a given ecological niche can occur through local adaptation, phenotypic plasticity, or a combination of these mechanisms. Evaluating the relative importance of these two mechanisms is needed to better understand the cause of intra specific polymorphism. In this study, we reared populations of Lake Whitefish (*Coregonus clupeaformis*) representing the’normal’ (benthic form) and the ‘dwarf’ (derived limnetic form) ecotypes in two different conditions (control and swim-training) to test the relative importance of adaptation and acclimation in the differentiation of traits related to swimming capacity. The dwarf whitefish is a more active swimmer than the normal ecotype, and also has a higher capacity for aerobic energy production in the swimming musculature. We hypothesized that dwarf fish would show changes in morphological and physiological traits consistent with reductions in the energetic costs of swimming and maintenance metabolism.

**Results:**

We found differences in traits predicted to decrease the costs of prolonged swimming and standard metabolic rate and allow for a more active lifestyle in dwarf whitefish. Dwarf whitefish evolved a more streamlined body shape, predicted to lead to a decreased drag, and a smaller brain, which may decrease their standard metabolic rate. Contrary to predictions, we also found evidence of acclimation in liver size and metabolic enzyme activities.

**Conclusion:**

Results support the view that local adaptation has contributed to the genetically-based divergence of traits associated with swimming activity. Presence of post-zygotic barriers limiting gene flow between these ecotype pairs may have favoured repeated local adaptation to the limnetic niches.

**Electronic supplementary material:**

The online version of this article (doi:10.1186/s12862-016-0732-y) contains supplementary material, which is available to authorized users.

## Background

Local adaptation occurs when individuals within a population evolve in response to selective pressure leading to increased fitness in their local environment relative to a foreign environment [1]. It is notoriously difficult to differentiate between local adaptation and environmentally induced plasticity in wild populations because phenotypic variation could be the product of either mechanism, or a combination of genetically based differentiation and plastic responses. Distinguishing between these two non-exclusive mechanisms is needed to better understand the cause of intra specific phenotypic polymorphism and the interaction of organisms with their environment [2–4]. This is particularly true for traits that commonly respond to a gradual change in the environment, a process known as acclimation (i.e. reversible, “physiological” phenotypic plasticity, or flexibility; [5]). Because it often increases survival in new environments, the process of acclimation is predicted to favour gene flow [6, 7], and hinder local adaptation [1]. Therefore, it is hypothesized that the extent of local adaptation should be inversely proportional to the level of gene flow between sympatric morphs exploiting different niches.

Northern freshwater fishes inhabiting postglacial environments show remarkable amounts of intra specific polymorphism [8–12] and numerous cases of parallel phenotypic evolution [13–17]. After the retreat of the Pleistocene ice sheets (~12,000 years ago), several species colonised newly formed lakes [18, 19], with free ecological niches allowing for phenotypic diversification [20, 21]. More specifically, sympatric ecotypes often show patterns of phenotypic differentiation associated with the benthic and limnetic niches [10, 11, 22–24]. A major difference between benthic and limnetic environments is the need to continually swim when foraging in the limnetic niche [24–27], as limnetic prey (i.e. zooplankton) show greater variation in abundance and distribution than benthic prey [24, 28]. The energy that is spent on swimming activity takes away from the energy available for other metabolic requirements such as growth, tissue maintenance and reproduction [29–31]. To maintain a balance between energy supply and demand and cope with the requirements for more active swimming in the limnetic niche, fish can adopt a number of strategies [25–27, 29, 30, 32]. These include decreasing the costs of swimming or the standard metabolic rate (i.e. the minimum metabolic rate needed to sustain life when animals are post-absorptive and inactive) [27, 31–33]. For instance, a common feature observed in limnetic morphs is a more hydrodynamic, slender body shape, which will diminish drag during steady swimming [24, 34, 35]. In contrast, benthic morphs often display deeper bodies, which enhance manoeuvrability and burst swimming capacity and increase foraging efficiency on benthic prey [24, 32, 33, 36]. Fish may also decrease the costs of maintenance metabolism by reducing the size of ‘metabolically-expensive’ organs. Indeed, studies of intra specific variation in standard metabolic rate often find positive correlations between standard/basal metabolic rate and the size of metabolically-expensive organs and a negative correlation with growth [31–33]. Brain and liver are two of the most metabolically-costly organs, so decreasing their size is predicted to reduce whole-animal metabolic maintenance cost [37–40]. A reduction of total gill surface area may also decrease whole organism energy expenditure because a larger gill surface area will lead to greater ion loss in freshwater, requiring fish to expend energy pumping ions to maintain osmoregulatory homeostasis [41]. These reductions in overall gill surface area may decrease oxygen uptake and hinder aerobic energy metabolism. However, fish normally use all of their gill area for oxygen uptake only during maximum aerobic swimming [42], which is not the case during limnetic foraging. Therefore, a decrease in gill surface area is predicted to be selected in freshwater environments when fish are energy limited, but oxygen is not limiting (i.e. normoxic waters). Overall, fish living in the limnetic niche are predicted to have more streamlined bodies, with smaller organ sizes (e.g. liver, brain, gills) compared to their benthic congeners, and thus save energy on maintenance costs and swimming which will allow for more energy to be spent on reproduction and growth (mainly in the white skeletal muscle) while actively foraging [29, 31].

Two Lake Whitefish (*Coregonus clupeaformis*) ecotypes are found in several postglacial lakes of the St. John River Drainage (Québec, Canada and Maine, USA) [43]. The ‘normal’ ecotype (Atlantic lineage) is specialized to a benthic niche while the repeatedly derived ‘dwarf’ ecotype (Acadian lineage) has colonised the free limnetic niche following secondary contact after the last glacial maximum [lineages were divided ~ 60,000 years ago or about 15,000 generations during the last Pleistocene glaciation and the secondary contact occurred ~ 12,000 years ago or about 3000 generations] [23, 44–47]. Reduced viability and sperm performance, reactivation of transposable elements and aneuploidy in hybrids are post-zygotic barriers that restrict gene flow between these ecotypes [48–52], in addition to ecological processes that may reduce the fitness of hybrids in natural conditions [11, 49]. As predicted by their ecological divergence, the limnetic ‘dwarf’ whitefish are more active swimmers and are younger at maturity (2–3 years vs 5 years or more) than normal fish, whereas normal whitefish grow more quickly, live longer, attain larger sizes, and have a higher condition factor than dwarf fish [23, 53, 54]. Studies examining the mechanisms underlying these differences in whole-animal performance have revealed that wild dwarf fish are more streamlined [55] and have a higher capacity for oxygen transport and use, which is required to fuel aerobic swimming (e.g. larger hearts, more red muscle, higher muscle mitochondrial content) [56–58]. Some traits related to maintenance and growth also follow predictions, as gill surface area in dwarf fish is smaller than in normal fish, but no differences in brain size are detected in wild caught fish [59]. Differences in gene expression between limnetic and benthic whitefish ecotypes have found extensive divergence in central energy metabolism (i.e. mitochondrial oxidative phosphorylation, citric acid cycle, glycolysis, glycogen metabolism, creatine phosphokinase). This metabolic divergence is predicted to underlie the observed trade-off in life-history traits, wherein enhanced survival via more active swimming is necessary for increased foraging and predator avoidance in the limnetic zone, but leads to energetic costs that translate into slower growth rates and reduced fecundity in dwarf relative to normal whitefish [23, 60–64].

However, the relative roles of acclimation and adaptation in the expression of these traits, including brain size, gill size and body shape is unclear because previous studies have all been performed on wild caught fish where dwarf and normal whitefish may be exposed to different environmental conditions [55, 59]. Moreover, juvenile and adult fish often show phenotypic plasticity in these traits in similar conditions [23, 65–67]. Therefore, the goal of this study is to test if variation in traits is due to acclimation and/or adaptation in controlled environments. We predicted that both mechanisms are used to compensate for the increase in energy allocation to locomotion in dwarf fish, but that adaptation should be a major mechanism underlying phenotypic divergence, considering the reproductive barriers previously documented between ecotypes pairs [11, 48–52]. We also further investigate the energetic strategies used by dwarf fish to inhabit the limnetic niche by measuring additional traits predicted to influence maintenance metabolism (i.e. liver size, liver metabolic capacity and a more detailed study of gill surface area).

## Methods

### Experimental setup

Crosses were produced from parents collected from Témiscouata (dwarf whitefish, Acadian lineage 47°36 N, 68°45 W) and Aylmer lakes (normal whitefish, Atlantic lineage, 45°50 N, 71°26 W) in Québec, Canada, caught in November 2011. It should be noted that these two populations are the only populations of whitefish ecotypes for which the breeding sites are known and crosses can be produced from. However, the divergence between these two allopatric ‘dwarf’ and ‘normal’ ecotypes is representative of the differentiation among other wild sympatric ecotype pairs. This is because transcriptomic comparisons among ecotypes show similar patterns of differentiation in these two populations and wild sympatric pairs of ecotypes [61]. As well, dwarf individuals from Témiscouata and normal individuals from Alymer lakes raised in the lab show heritable differentiation in behavioural traits in accordance with the different life history traits expected between limnetic and benthic fishes [53] and show the same postzygotic isolation mechanisms described above.

**Fig. 1:**
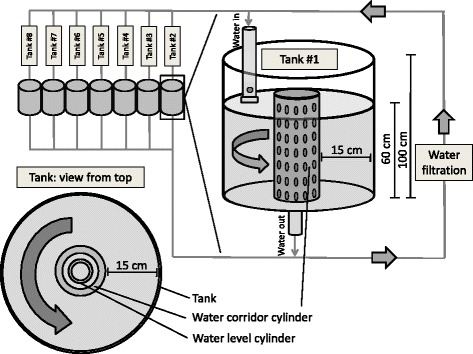
Schematic representation of the experimental setup testing the effect of ecotypes (dwarf vs. normal) and treatment (control vs. swim-training) in Lake Whitefish. Water velocity was set at higher speeds in half of the tanks and 8 individual of each ecotypes were present in each tanks to ensure blind sampling (see text for details)

The fish used in this study are the same as those used in a recently conducted experiment [58]. Briefly, gametes from dwarf and normal whitefish were collected in the field and brought to the Laboratoire de Recherche en Sciences Aquatiques (LARSA, Université Laval) for artificial fertilization following [63]. Gametes from multiple females and males were mixed (dwarf: seven females with seven males; normal: nine females with 14 males). We reared crosses in a fresh-water, flow through system under identical temperatures, lighting schedules, water velocities, and feeding regimes for the first 16 months. The experiment started at ~ 17 months of age and finished 6 months later before fish began to invest energy in reproduction. Since no mature gonads were observed during sampling, acclimation and not differences in maturation should have caused the observed treatment effects (if any) during the experiment. A total of 127 fish (63 dwarf and 64 normal) were separated into eight 1 m tall × 50 cm diameter circular tanks (Fig. 1). To ensure random sampling, each tank contained an equivalent number of dwarf and normal whitefish of similar size and weight to obtain an overall equivalent biomass. We re-identified each fish as being dwarf or normal *a posteriori* by using a diagnostic mitochondrial RFLP assay after sampling [58, 68]. Half of the tanks (which we refer to as “swimming tanks”) were set with a water current of 10 cm/s for 6 h/day. We chose experimental speeds to match the natural activity level expected in the limnetic niche (constant and aerobic swimming during foraging). This was confirmed by our observations that i) at higher water velocities it was difficult for some individuals to maintain their position in the current and ii) previous studies show that Lake Whitefish have a lower prolonged swimming capacity than other salmonids [69, 70]. The other tanks (which we refer to as “control tanks”) had only a very low water current (<0.5 cm/s) required to allow for flow-through conditions, 24 h/day. At the end of the experiment, no difference in mass was observed between ecotypes [58]. More details on the rearing conditions are available in Dalziel et al. [58]. All protocols were approved by Université Laval’s animal care committee (Protocol 82178).

### Phenotypic measurements

**Fig. 2:**
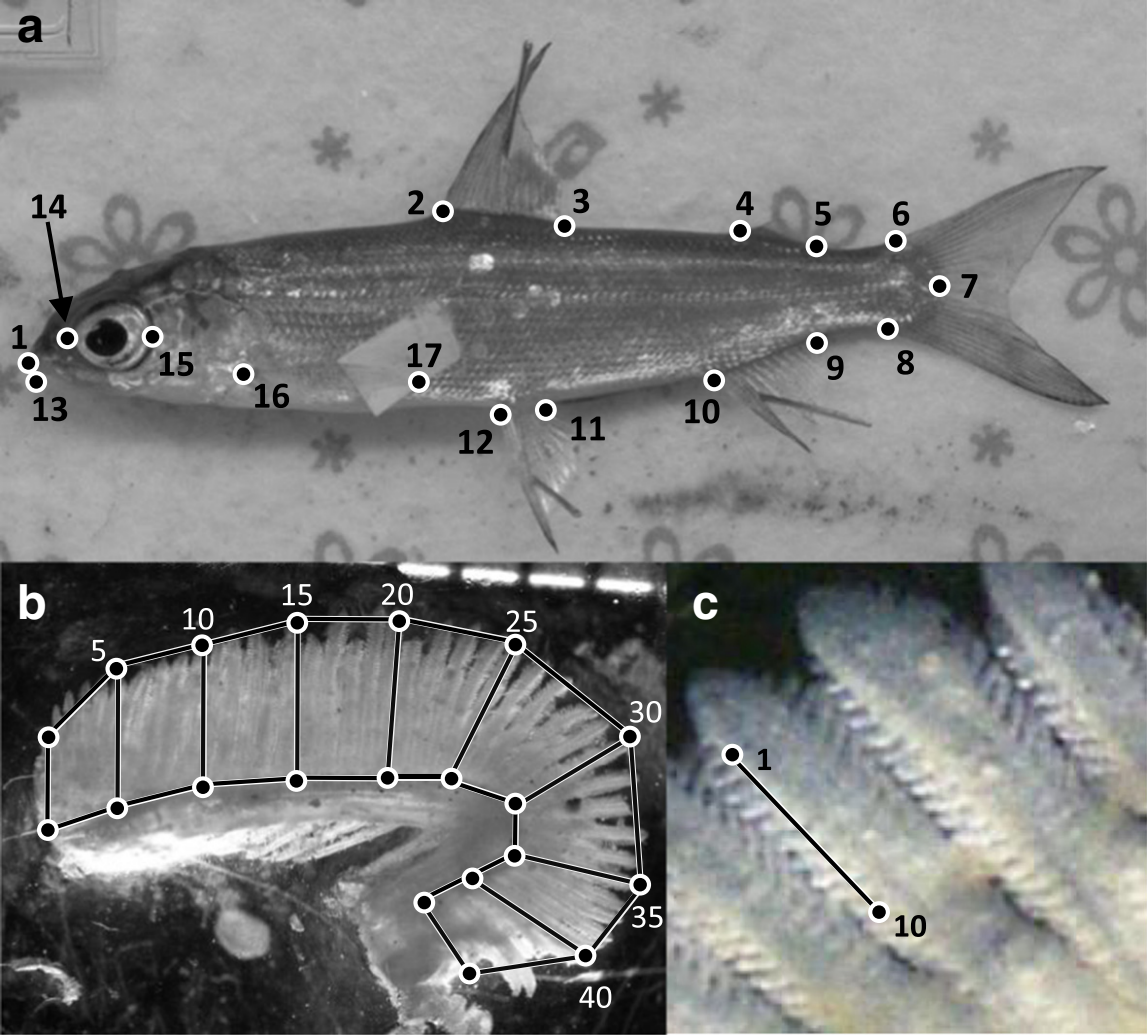
**a** Positions of 17 landmarks on Lake Whitefish (1: snout; 2–3: start and end of the dorsal fin; 4–5: start and end of the adipose fin; 6-7-8: top, end and bottom of the caudal peduncle; 9–10: end and start of the anal fin; 11–12: end and start of the pelvic fin; 13: end of maxilla; 14–15: eyes; 16–17: start and end of the pectoral fin). **b** Example of measurement estimating gill surface area (first left arch), including the number of filaments and average length of filaments. **c** Example of measuring space between lamellae

Shape analyses were based on geometric landmark coordinates [71]. Seventeen landmarks were digitized on each image (Fig. 2a) using tpsDig v2.16 [72]. We chose landmarks to reflect characteristics predicted to be associated with fish locomotion [25, 26, 73]. Fish were sacrificed with pithing using a needle followed by cervical dislocation. Immediately after fish were euthanized, digital photographs of the left side of the fish were taken with a Nikon Coolpix P7700 camera to avoid shape deformation that can be associated with preservation [65, 74]. Fish were repositioned and a second image was captured to estimate measurement error, adapted for Procrustes data [75, 76]. A Partial Generalized Procrustes Analysis superimposition was first conducted to preserve information on shape differences among fish and to remove information unrelated to shape (i.e. scale, position, and orientation) [77, 78]. Fish shape was estimated from the superimposed coordinates projected on principal component analysis (PCA) using the MorphoJ software v1.06 [79]. We used the wireframe graph option of MorphoJ to display shape change on PC-axes considered informative, based on a broken-stick distribution [80].

After the images were captured, wet brain and liver mass were determined and gills were preserved in 4 % formaldehyde. The first arch of the left gill was laid flat on a microscope slide and photographed. We counted the number of filaments and estimated the average length of filaments, total length of filaments, average space between lamellae, number of lamellae and total hemibranch area using ImageJ [81]. The two latter estimates were our gill area proxies. More precisely, the average filament length was estimated by measuring each fifth filament and divided the total by the number of filaments measured (Fig. 2b). These points were also used to obtain shape and measure of hemibranch area (Fig. 2b). Total filament length was obtained by multiplying the average filament length and number of filaments. Average space between lamellae was estimated by the distance between ten lamellae, and measured five times on different parts of the gill to control for measurement error (Fig. 2c). Finally, the total number of lamellae was calculated by dividing the total filament length by the average space between lamellae and multiplied by ten (since average space between lamella was measured on 10 lamellae).

To study the metabolic capacity of the liver, we measured the activity of COX (cytochrome c oxidase, EC 1.9.3.1, complex IV in the electron transport chain, and found on the inner mitochondrial membrane) and CS (citrate synthase, EC 2.3.3.1, a citric acid cycle enzyme found in the mitochondrial matrix) per gram of liver tissue and calculated enzyme activities for the entire liver by multiplying this by total liver mass. COX and CS activities are indicators of mitochondrial content and should reflect the metabolic capacity of the liver [82]. To do this, we re-weighed frozen liver (g), and immediately added 20 volumes of chilled homogenization buffer (50mmoll −1 hepes, 1mmoll −1 EDTA and 0.1 % Triton X-100; pH7.4) in 4 ml Wheaton glass homogenizers kept on ice. Enzyme activities were measured on whole-cell extracts at 25 °C using non-limiting substrate concentrations outlined by [58].

### Statistical tests

We first tested for a relationship between each trait and body mass and used residuals to remove allometric effects when there was a significant correlation (*P* < 0.05). To test the effect of ecotype (fixed effect, dwarf or normal whitefish) and treatment (fixed effect, control or swim) we ran mixed effects linear models using the ‘nlme’ package in R v 3.1.1 with tanks nested as a random effect and individual fish nested within tanks (Two-way nested ANOVAs). Acclimation was defined as a significant fixed-effect of treatment (e.g. both ecotypes display a similar direction and extent of plasticity) or a significant interaction effect (e.g. only one ecotype is plastic or ecotypes display different directions and/or extents of plasticity) and a genetic control was defined by similar criteria (fixed effect of ‘ecotypes’ or a significant interaction indicating evolutionary variation in plasticity). We also performed a multivariate analysis to determine if traits measured in this study could differentiate our four experimental groups (i.e. ecotype X treatment: dwarf/control - dwarf/swim - normal/control - normal/swim) and determine which traits best differentiated them. To do this we performed a linear discriminant function analysis (DFA) on mean value specific to each ecotypes for each tank. All variables were standardized and the function ‘lda’ in R software v 3.1.1 was used to perform the DFA.

## Results

The estimated variance between the two body shape measures for the same individual, measured from two independent pictures taken after repositioning the fish, was on average 2.0 % of the variance between any two different individuals. This indicates that the variation among individuals is 50 times higher than the variation produced between two pictures of the same individual (i.e. measurement error). The differences among individuals explained a significant portion of the shape variation (MANOVA; d.f. = 126; approx. F = 4.43; *P* < 0.001). Therefore, the shape differentiation observed among experimental groups (if any) should not have been produced by fish manipulation and/or landmark positioning.

**Fig. 3:**
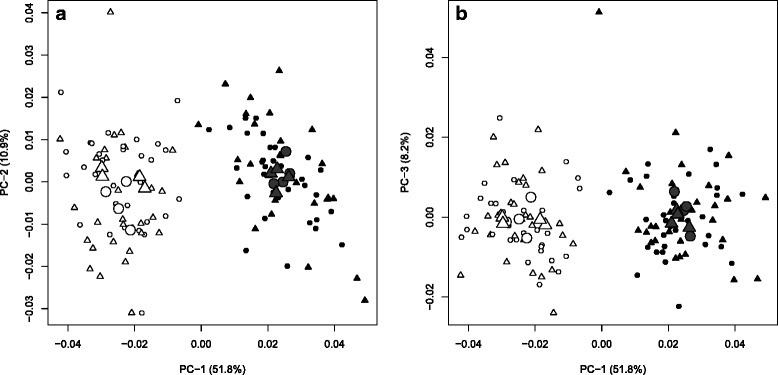
Shape comparisons between normal (*black*) and dwarf (*white*) Lake Whitefish in control (*circle*) and swim-training (*triangle*) treatments in three PC-axes considered informative based on a broken-stick distribution (**a**) PC-1 and PC-2 and (**b**) PC-1 and PC-3). The larger symbols correspond to the averages for each tank

**Fig. 4:**
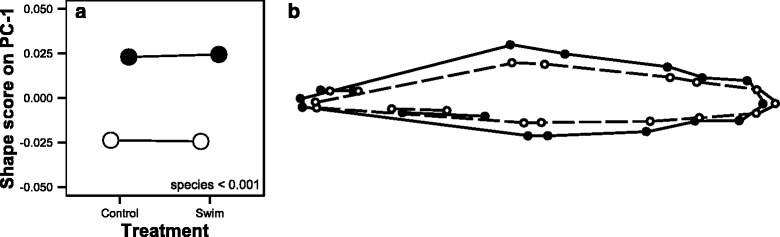
**a** Shape variation along PC-1 of swim-trained and control dwarf (*white circle*) and normal (*black circle*) Lake Whitefish. The effect of treatment, ecotypes and/or their interaction are noted when *P*-values < 0.05. **b** Wireframe graph displaying shape changes on the first PC axis between ecotypes: dwarf (*white dots* and *dashed lines*) and normal (*black dots* and *straight lines*) whitefish

Based on the broken-stick distribution, the first three shape PCs were considered informative and represented respectively 51.8 %, 10.9 % and 8.2 % of fish shape variation (Fig. 3a, b). Dwarf and normal whitefish were clearly separated on the first PC (Fig. 3a, b). This was confirmed by a two-way nested ANOVA since the effect of ecotype significantly explained fish shape PC-1 variation (*P* < 0.001; Fig. 4a). No effect of ecotype was observed on PCs 2 and 3 and no effect of treatment or interaction was observed for any informative PCs (Fig. 3a, b; Additional file 1). Dwarfs differ from the normal whitefish in shape because they have a more slender body, shorter pectoral fins, longer caudal peduncles and shorter dorsal fin bases (Fig. 4b). Finally, shape differentiation on PCs 2 and 3 shows variation that did not seem to be linked to functional morphology associated with swimming (Additional file 2), thus these two PCs have been removed from the subsequent multivariate analysis.

**Fig. 5:**
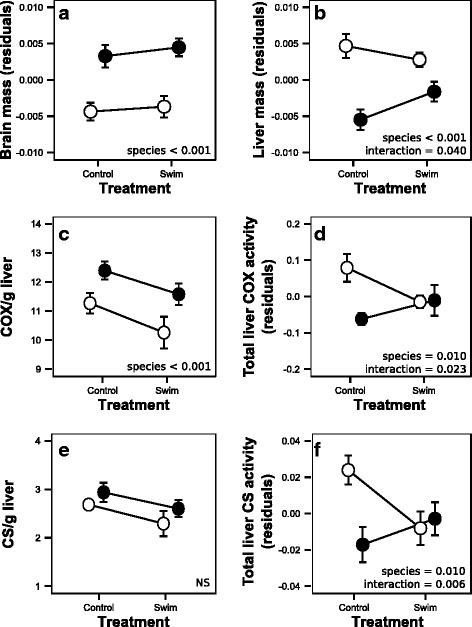
Brain mass (**a**), liver mass (**b**), COX/g liver (**c**), total liver COX activity (**d**), CS/g liver (**e**) and total liver CS activity (**f**) of swim-trained and control dwarf (*white circle*) and normal (*black circle*) Lake Whitefish. The effect of treatment, ecotypes and/or their interaction are noted when *P*-values < 0.05. NS was noted when no significant differences were observed

Dwarf whitefish brains (size adjusted) were smaller than those from normal fish (*P* < 0.001; Fig. 5a). However, dwarf whitefish livers were larger (*P* < 0.001; Fig. 5b) and an interaction between ecotypes and treatment was also observed for liver mass (*P* = 0.040; Fig. 5b). Interestingly, dwarf whitefish showed a trend towards a decreased liver mass in the swimming treatment, while normals showed the inverse (Fig. 5b). As COX and CS activities are indicators of mitochondrial content [82] and should reflect the metabolic capacity of the liver, we further investigated how liver mass may influence whole-animal metabolism by measuring activity of these two mitochondrial enzymes. A significantly higher COX activity per gram was observed for the normal ecotype (*P* < 0.001; Fig. 5c). No significant difference was observed for CS activity per gram despite a nearly significant trend among ecotypes (*P* = 0.069; Fig. 5e; Additional file 1). After calculating total liver enzyme activity, we observed an effect of ecotype for both COX and CS activity (*P* = 0.010; Fig. 5d; *P* = 0.010; Fig. 5f) and an interaction (*P* = 0.023; Fig. 5d; *P* = 0.006; Fig. 5f). Similar to the liver mass, total liver COX and CS activity decreased in the swim treatment group for dwarf whitefish, but increased for normal fish after swim-training.

**Fig. 6:**
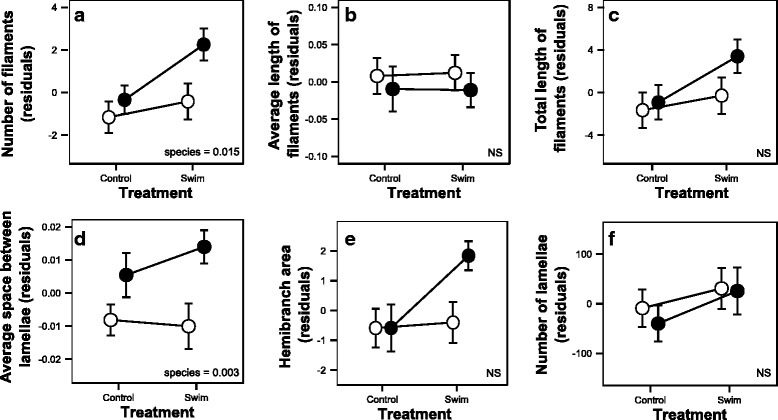
Measures to calculate gill surface area (number of filaments (**a**), average length of filaments (**b**), total length of filaments (**c**), average space between lamellae (**d**), hemibranch area (**e**) and number of lamellae (**f**)) of swim-trained and control dwarf (*white circle*) and normal (*black circle*) Lake Whitefish. The effect of treatment, ecotypes and/or their interaction are noted when *P*-values < 0.05. NS was noted when no significant differences were observed

A significant effect of ecotype was observed for the number of gill filaments (*P* = 0.015; Fig. 6a) and average space between gill lamellae (*P* = 0.003; Fig. 6d), which were both higher in normal fish compared to dwarf fish. No significant effect of ecotype, treatment or their interaction was observed for the average length of filaments, total length of filaments and number of lamellae (Fig. 6b, c and f). However, a nearly significant interaction (*P* = 0.066) was observed for hemibranch area, which increased in only the normal whitefish after swim-training (Fig. 6e). All results for our univariate analyses are presented in the Additional file 1.

**Fig. 7:**
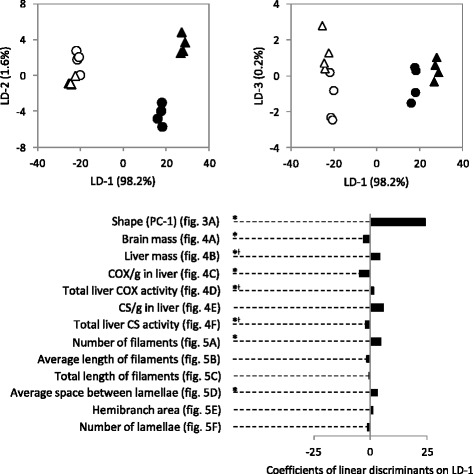
Top- DFA discriminating fish by group (dwarf-control: *white circle*; dwarf-swim trained: *white triangle*; normal-control: *black circle*; normal-swim trained: *black triangle*) with the percentage of variation explained for each of the three axes. Each point represent one of the eight tanks. Bottom- factor loadings contributing on the first ld for each traits measured (see Figs. 4, 5 and 6)

The DFA clearly differentiated dwarf and normal whitefish on the first linear discriminant (ld), accounting for 98.2 % of the variation (Fig. 7). Shape was the most important factor discriminating groups on the first ld according to a comparison of coefficient of linear discriminations (24.33 for shape *vs* less than 5.00 for all others traits; Fig. 7). The two last lds represented a total 1.8 % of the variation with no clear pattern, despite possible discrimination of normal whitefish by treatment on the second ld (Fig. 7).

## Discussion

We found a significant effect of ecotype for eight traits that are predicted to mediate energetic trade-offs between dwarf and normal whitefish by contributing to reductions in the cost of prolonged swimming (body streamlining) and standard metabolic rate (liver, brain and gill size). Three of these eight traits (all linked to the liver: liver mass, total liver COX activity, total liver CS activity) also showed a significant interaction between ecotype and environment, but no treatment effect was observed. Based on ecological and functional expectations, only body shape and brain mass match our predictions for reductions in swimming and metabolic maintenance costs in the more active dwarf whitefish, while liver mass varied in the opposite direction to that which was predicted. In accordance with the presence of postzygotic barriers between these ecotypes [11, 48–52], our results indicate that adaptation (more than acclimation) is the major mechanism allowing specialisation to the limnetic niche. Furthermore, fish shape was the trait that best differentiated ecotypes in the multivariate analysis, suggesting that the evolution of fish shape is of major importance in the colonisation of a new trophic niche which requires a more active lifestyle. In contrast, traits associated with liver metabolism and gill size do not match our predictions. This could be the result of either genetic or functional constraints (i.e. limitations on how traits could evolve due to functional roles in other performance traits/biochemical pathways that strongly select against change) or stochastic evolutionary processes (e.g. genetic drift). Finally, the three traits associated with liver metabolism showing ecotype–environment interactions suggest that liver energy metabolism is more responsive to swimming activity than the other traits measured in this study.

### Local adaptation vs. Acclimation in Lake Whitefish ecotypes

Three traits had significant ecotype–environment interactions and all were associated with liver metabolism (liver mass, total COX activity and total CS activity). In comparison, the genetic and environmental bases of traits related to oxygen uptake, transport and use in the same fish were also tested [58]. Eight traits related to ventricle size and metabolism and white muscle mitochondrial respiration displayed evidence of acclimation for at least one ecotype. In contrast, twice (*n* = 15) as many traits, including hematocrit, skeletal muscle mitochondrial function and the activities of multiples enzyme displayed a strong genetic basis and limited acclimation. This suggests that acclimation to high swimming activity leads to changes in liver metabolism, ventricle size and mitochondrial respiration, but that traits related to body shape, brain size, gill size, hematocrit and skeletal muscle mitochondrial content are mainly under genetic control. Similar experiments in other salmonids commonly find a significant effect of exercise on morphological and physiological traits (e.g. heart size and mitochondrial enzyme activities, skeletal muscle metabolic enzyme activities and body shape) [34, 65, 83–85]. Here, we chose a water velocity between one and two body lengths per second to emulate the constant aerobic swimming required for the exploitation of the limnetic niche (zooplankton are more aggregate and dispersed than benthic prey in lakes) [28]. This swimming speed should emulate the cruising speeds of many fish species in the wild [86]. As well, our experimental fish did not respond well to higher water velocities, so we did not increase swimming speeds to the levels that were used in other studies with salmonid fishes [70]. Our fish were swim-trained for six months, which is normally sufficient to induce a plastic response [34, 65, 85]. For instance, a significant effect of swim-training on body shape after training at a similar water velocity was found in the benthic fish *Salaria fluviatilis* after only 28 days [65]. Together, these data suggest that Lake Whitefish acclimation to swim-training is relatively subtle in comparison to evolutionary divergence. This hypothesis is also supported by the first axis of the discriminant function analysis that accounts for 98.2 % of the variation among groups and strongly differentiates ecotypes but not treatments. Lake Whitefish ecotypes are known to have the strongest reproductive barrier among all studied species-pairs of northern temperate fishes undergoing ecological speciation [11], and our results are consistent with the hypothesis that strong reproductive barriers are linked to the accumulation of adaptive genetic divergence between sympatric ecotypes [87, 88]. Admittedly, our results do not exclude the possibility that other environmental variables (e.g. diet, predation, temperature, dissolved oxygen content), which vary between benthic and limnetic niches could induce a plastic response on these traits, nor can we rule out the presence of developmental plasticity at an earlier life stage.

### Limits of the experimental setup

Comparing organisms reared in different controlled environments allow biologists to test for the presence of genetic differentiation and local adaptation [1]. Differentiation observed in a trait of interest among environmental treatments indicates phenotypic plasticity in this trait, differentiation in a trait among ecotypes in the same environment indicates genetic divergence, and differential responses to environments among ecotypes suggests that phenotypic plasticity has evolved differently [3]. To rigorously test for genetic differentiation, second-generation offspring reared in a common garden must be used in the controlled experiment, since long-term phenotypic plasticity produced by prior parental exposure to different environments is possible [3, 89]. In the present study, parents could not be chosen from grandparents reared in a common garden because of the long generation time of the Lake Whitefish (2–3 years for the dwarf and 5 years or more for the normal whitefish) and the difficulty of maintaining enough individuals for a second round of artificial fertilization without losing a substantial amount of genetic diversity. Despite the fact that long term environmental effects cannot be excluded, we clearly ruled out short term acclimation as a major mechanism leading to traits variation. In addition, previous studies comparing reciprocal hybrid crosses (dwarf female X normal male; normal female X dwarf male) found no significant differences in swimming activity among crosses [53]. These data suggest that the parent of origin does not have a significant effect on these performance traits and that, if present, parental effects are equally transmitted by males and females [53]. Finally, the findings from this study extend mainly to the comparison of this particular set of dwarf and normal whitefish.

### Decreasing the cost of swimming

When a species begins to forage on a new resource, a first and crucial step in successful foraging is having the ability to reach this new resource, which comes at an energetic cost of transporting the body to the resource’s location [86, 90, 91]. Fish shape influences locomotion, and ultimately foraging efficiency. Thus, it is predicted to be under selection when major changes in diet occur that require a change in swimming mode [25–27, 32]. We found that dwarf whitefish have evolved a more slender body with smaller pectoral fins. Such shape differentiation is generally associated with the limnetic niche in salmoniform swimmers as it decreases drag, and allows fish to expend less energy foraging on dispersed zooplankton [25–28, 32]. In contrast, a stouter body and longer pectoral fins are expected to increase manoeuvrability and are predicted to be beneficial when foraging on benthic organisms [24–27, 32]. Fish shape was the trait that best discriminated our four experimental groups (five times more than any other trait), suggesting the importance of shape evolution during the colonisation of the limnetic niche. Because no effect of treatment was observed on body shape, the use of the benthic niche by a dwarf individual or the limnetic niche by a normal individual would likely result in a decrease in foraging efficiency. Such adaptive phenotypic divergence in foraging efficiency has been observed in the European Whitefish ecotypes as well [92]. As discussed above, a long term environmental effect, such as parental effects, could not be ruled out by our experimental setup. However, similar shape differentiation in wild fish and a signal of selection on shape-QTL (Quantitative Trait Loci) have previously been documented, indicating that shape is genetically based and reflects adaptive divergence [55].

### Diminishing costs of organ maintenance metabolism

With respect to maintenance metabolism, including the costs of tissue maintenance, reproduction and growth, we predicted that dwarf whitefish would show reductions in liver and brain size as both are metabolically expensive organs to maintain [37–39]. Reductions in the size of these organs may allow for an increase in energy available for foraging in the limnetic niche. As predicted, dwarf whitefish had a smaller brain mass than the normal ecotype, suggesting that a reduction in energy expenditure could be used to divert additional energy towards locomotion. However, no parallel trend in brain mass differentiation was observed among wild ecotypes of Lake Whitefish [59]. Nevertheless, a bigger or a smaller brain in dwarf whitefish could be observed when considering a single sympatric pair [59]. It is possible that other ecological conditions induce a plastic response in brain mass in wild Lake Whitefish [66, 93–95]. For example, changes in brain structures were found in fish actively escaping predators [66, 93] and it is thought that the smaller dwarf whitefish suffer from higher predation pressure than normal fish [46, 53, 54, 96]. Further research specifically addressing brain structure differentiation will be needed to unravel this organ’s role in the origin of the dwarf ecotype.

Contrary to our predictions, we found that the liver was larger in dwarf whitefish. We further investigated this finding by testing if the maintenance costs of liver tissue might vary among ecotypes. A higher mitochondrial content is predicted to be associated with a higher standard metabolic rate because of increases in membrane density and transmembrane ion gradient maintenance costs [97, 98]. Indeed, variation in liver mitochondrial content (COX and CS enzyme activity) correlates positively with differences in whole-fish standard metabolic rate among species, while total liver size does not [40]. As predicted, we found that the enzyme activities of COX and CS were higher in normal whitefish per gram of liver indicating a higher mitochondrial content per gram of tissue than for dwarf fish. However, total liver enzyme activity, which is a function of both activity and liver size, remained higher in dwarf than normal fish under control conditions, opposite to predictions. Interestingly, there was an interaction between ecotype and environment for both enzymes such that when fish were subjected to swim-training, differences in overall liver enzyme activity were no longer found. This suggests that potential reduction in liver size in dwarf whitefish is constrained by the many other physiological functions this organ is involved in [99]. For example, the higher food consumption rate of the dwarf whitefish could necessitate a relatively larger liver to support increases in gluconeogenesis, blood filtration and waste management [30, 61].

We also predicted dwarf fish to have a smaller total gill surface area because a reduction in ion loss in these freshwater fish could decrease whole organism energy expenditure [41, 42]. We observed an ecotype effect that revealed fewer gill filaments and smaller space between lamellae in the dwarf whitefish. Fewer gill filaments matches our prediction but smaller space between lamellae does not. Since no significant differentiation was observed in the number of lamellae, the variation in filament number and lamellar spacing negate each other leading to similar gill surface areas. In contrast, a nearly significant ecotype-environment interaction in hemibranch area was also observed. However, this trait does not take into account differences in space between lamellae when estimating gill surface area. The similar total gill surface areas in dwarf and normal fish suggests that differences in underlying gill morphological traits (e.g. lamellar spacing and number of filaments) is likely due to stochastic evolutionary processes [23]. In wild fish, there were differences in hemibranch area, hemibranch perimeter, number of filaments, average length of filaments and total length of filaments, leading to a slightly, but not significantly, larger gill surface area in normal whitefish, but space between lamellae was not measured [59]. Here, we found that space between lamellae may have contributed to this difference between wild ecotypes and that there are no differences in overall gill surface area among lab-reared ecotypes when it is taken into account.

## Conclusion

We found differences in a number of morphological and physiological traits predicted to respond to the more active and energy demanding lifestyle of the dwarf fish, associated with occupying the limnetic niche. In combination with Dalziel et al. [58], a total of 23 traits related to body shape, brain size, liver size, gill surface area, ventricle mass, ventricle and skeletal muscle metabolism, mitochondrial function and hematocrit show a predominant genetic basis for the variation observed in these traits between dwarf and normal Lake Whitefish. The two studies together also revealed that eleven traits related to ventricle mass, mitochondrial function and liver mass show an induced-environmental response for at least one ecotype. Overall, these data suggest that local adaptation (more than acclimation) is the major mechanism underlying the divergence of swimming activity between dwarf and normal whitefish, suggesting that there is a low probability for fish to switch trophic niches within a lifetime in the wild. The well-documented reproductive barriers between these ecotypes and genetically based differences in several behavioural, physiological and morphological traits underlying differences in swimming activity also support this hypothesis [48–55].

## Additional files

Additional file 1: Details of the two-way nested ANOVAs on the 13 traits measured and graphically presented in the Figs. 4, 5 and 6. (DOCX 20 kb) Additional file 2: Wireframe graph displaying shape change on the second PC axis (top) and third PC axis (bottom): dwarf (white dot and dash line) and normal (black dot and straight line) whitefish. (PDF 7 kb)
