# Supplementary material for: Adaptation and acclimation of traits associated with swimming capacity in Lake Whitefish (coregonus clupeaformis) ecotypes
Source: BMC Evol Biol. 2016 Aug 11;16:160. doi: 10.1186/s12862-016-0732-y (PMC4982116; doi:10.1186/s12862-016-0732-y)
Supplement: Additional file 1: — Details of the two-way nested ANOVAs on the 13 traits measured and graphically presented in the Figs. 4, 5 and 6. (DOCX 20 kb) [file 12862_2016_732_MOESM1_ESM.docx]

**Supplementary material 1.**

| **SHAPE (Fig. 4)** |  |  |  |  |
| --- | --- | --- | --- | --- |
| **PC1** | **numDF** | **denDF** | **F-value** | **p-value** |
| (Intercept) | 1 | 115 | 0.0006 | 0.9811 |
| treatment | 1 | 6 | 0.0798 | 0.787 |
| species | 1 | 115 | 803.5063 | <.0001*** |
| treatment:species | 1 | 115 | 0.3465 | 0.5573 |
| **PC2** | **numDF** | **denDF** | **F-value** | **p-value** |
| (Intercept) | 1 | 115 | 0 | 1 |
| treatment | 1 | 6 | 0.821324 | 0.3997 |
| species | 1 | 115 | 2.504703 | 0.1163 |
| treatment:species | 1 | 115 | 3.147039 | 0.0787 |
| **PC3** | **numDF** | **denDF** | **F-value** | **p-value** |
| (Intercept) | 1 | 115 | 0 | 1 |
| treatment | 1 | 6 | 0.58955 | 0.4717 |
| species | 1 | 115 | 0.598701 | 0.4407 |
| treatment:species | 1 | 115 | 0.135385 | 0.7136 |
| **Brain - Liver - Enzyme activity (Fig. 5)** | |  |  |  |
| **Brain** | **numDF** | **denDF** | **F-value** | **p-value** |
| (Intercept) | 1 | 110 | 0.00001 | 0.9972 |
| treatment | 1 | 6 | 0.2377 | 0.6432 |
| species | 1 | 110 | 35.15331 | <.0001*** |
| treatment:species | 1 | 110 | 0.0301 | 0.8626 |
| **Liver** | **numDF** | **denDF** | **F-value** | **p-value** |
| (Intercept) | 1 | 113 | 0 | 1 |
| treatment | 1 | 6 | 0.49645 | 0.5075 |
| species | 1 | 113 | 28.12689 | <.0001*** |
| treatment:species | 1 | 113 | 4.30921 | 0.0402* |
| **COX/g** | **numDF** | **denDF** | **F-value** | **p-value** |
| (Intercept) | 1 | 42 | 881.2506 | <.0001 |
| treatment | 1 | 6 | 0.8216 | 0.3996 |
| species | 1 | 42 | 21.4137 | <.0001*** |
| treatment:species | 1 | 42 | 0.5385 | 0.4671 |
| **Total COX** | **numDF** | **denDF** | **F-value** | **p-value** |
| (Intercept) | 1 | 41 | 0.002166 | 0.9631 |
| treatment | 1 | 6 | 0.255789 | 0.6311 |
| species | 1 | 41 | 7.208749 | 0.0104* |
| treatment:species | 1 | 41 | 5.568479 | 0.0231* |
| **CS/g** | **numDF** | **denDF** | **F-value** | **p-value** |
| (Intercept) | 1 | 42 | 378.7023 | <.0001 |
| treatment | 1 | 6 | 1.4056 | 0.2806 |
| species | 1 | 42 | 3.4714 | 0.0694 |
| treatment:species | 1 | 42 | 0.2317 | 0.6328 |
| **Total CS** | **numDF** | **denDF** | **F-value** | **p-value** |
| (Intercept) | 1 | 41 | 0.004748 | 0.9454 |
| treatment | 1 | 6 | 0.498496 | 0.5066 |
| species | 1 | 41 | 7.340606 | 0.0098** |
| treatment:species | 1 | 41 | 8.322867 | 0.0062** |
| **Gills (Fig. 6)** |  |  |  |  |
| **n. of filaments** | **numDF** | **denDF** | **F-value** | **p-value** |
| (Intercept) | 1 | 57 | 12043.52 | <.0001 |
| treatment | 1 | 6 | 3.817 | 0.0986 |
| species | 1 | 57 | 6.29 | 0.015* |
| treatment:species | 1 | 57 | 1.314 | 0.2565 |
| **av. length of filaments** | **numDF** | **denDF** | **F-value** | **p-value** |
| (Intercept) | 1 | 57 | 0.020004 | 0.888 |
| treatment | 1 | 6 | 0.161751 | 0.7015 |
| species | 1 | 57 | 0.505908 | 0.4798 |
| treatment:species | 1 | 57 | 0.003326 | 0.9542 |
| **av. length between 10 lamallea** | **numDF** | **denDF** | **F-value** | **p-value** |
| (Intercept) | 1 | 57 | 0 | 1 |
| treatment | 1 | 6 | 0.241629 | 0.6405 |
| species | 1 | 57 | 9.609489 | 0.003** |
| treatment:species | 1 | 57 | 0.765953 | 0.3851 |
| **Gill area** | **numDF** | **denDF** | **F-value** | **p-value** |
| (Intercept) | 1 | 57 | 0.03612 | 0.8499 |
| treatment | 1 | 6 | 1.304045 | 0.297 |
| species | 1 | 57 | 2.744211 | 0.1031 |
| treatment:species | 1 | 57 | 3.503989 | 0.0664 |
| **Total length of filaments** | **numDF** | **denDF** | **F-value** | **p-value** |
| (Intercept) | 1 | 57 | 0 | 1 |
| treatment | 1 | 6 | 2.842928 | 0.1428 |
| species | 1 | 57 | 1.630193 | 0.2069 |
| treatment:species | 1 | 57 | 0.797265 | 0.3757 |
| **Number of lamallea** | **numDF** | **denDF** | **F-value** | **p-value** |
| (Intercept) | 1 | 57 | 0 | 1 |
| treatment | 1 | 6 | 1.697653 | 0.2404 |
| species | 1 | 57 | 0.221736 | 0.6395 |
| treatment:species | 1 | 57 | 0.100666 | 0.7522 |
| * *P* < 0.05; ** *P* < 0.01; *** *P* < 0.001 | |  |  |  |
