# Supplementary figures and images for: Adaptation and acclimation of traits associated with swimming capacity in Lake Whitefish (coregonus clupeaformis) ecotypes
Source: BMC Evol Biol. 2016 Aug 11;16:160. doi: 10.1186/s12862-016-0732-y (PMC4982116; doi:10.1186/s12862-016-0732-y)

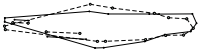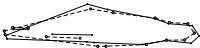

Supplement: Additional file 2: — Wireframe graph displaying shape change on the second PC axis (top) and third PC axis (bottom): dwarf (white dot and dash line) and normal (black dot and straight line) whitefish. (PDF 7 kb) [file 12862_2016_732_MOESM2_ESM.pdf]
